# The Dynamic Modulation Doping Effect of Gas Molecules on an AlGaN/GaN Heterojunction Surface

**DOI:** 10.3390/nano14141211

**Published:** 2024-07-16

**Authors:** Ying Ma, Lin Shi, Liang Chen, Cai Chen, Yifang Hong, Hua Qin, Xiaodong Zhang, Yi Cui, Hongzhen Lin, Zhiqun Cheng, Fan Zhang, Linfeng Mao, Yong Cai

**Affiliations:** 1 School of Nano-Tech and Nano-Bionics, University of Science and Technology of China, Hefei 230026, China; 2 Key Lab. of Nanodevices and Applications, Suzhou Institute of Nano-Tech and Nano-Bionics, Chinese Academy of Sciences (CAS), Suzhou 215123, China; 3 School of Materials Science and Engineering, Yancheng Institute of Technology, Yancheng 224051, China; linshi@ycit.edu.cn; 4 School of Electronics and Information, Hangzhou Dianzi University, Hangzhou 310018, China; 5 Nanofabrication Facility, Suzhou Institute of Nano-Tech and Nano-Bionics, Chinese Academy of Sciences (CAS), Suzhou 215123, China; 6 I-Lab, Vacuum Interconnected Nanotech Workstation (Nano-X), Suzhou Institute of Nano-Tech and Nano-Bionics, Chinese Academy of Sciences (CAS), Suzhou 215123, China; 7 School of Computer & Communication Engineering, University of Science & Technology Beijing, Beijing 100083, China

**Keywords:** AlGaN/GaN heterojunction, Dynamic doping, 2DEG

## Abstract

AlGaN/GaN high-electron-mobility transistors (HEMTs) are widely used in high-frequency and high-power applications owing to the high two-dimensional electron gas (2DEG) concentration. However, the microscopic origin of the 2DEG remains unclear. This hinders the development of device fabrication technologies, such as threshold voltage modulation, current collapse suppression, and 2DEG concentration enhancement technologies, as well as AlGaN/GaN sensors with very high sensitivity to polar liquids. To clarify the 2DEG microscopic origin, we studied the effects of gas molecules on AlGaN/GaN surfaces through various experiments and first-principles calculations. The results indicated that the adsorption of gas molecules on the AlGaN/GaN surface is an important phenomenon, clarifying the microscopic origin of the 2DEG. This study elucidates the properties of AlGaN/GaN heterojunctions and promotes the development of new fabrication technologies for AlGaN/GaN devices.

## 1. Introduction

In recent decades, there have been rapid advancements in communications, computing, and artificial intelligence; consequently, integrated circuit systems have undergone significant miniaturization, and their power densities and operating frequencies have increased. AlGaN/GaN high-electron-mobility transistors (HEMTs) have been widely used in high-frequency and high-power applications owing to their high breakdown voltages, high working temperatures, and chemical inertness [1,2,3,4,5]. Furthermore, these HEMTs have a high two-dimensional electron gas (2DEG) concentration of >10^13^ cm^−2^ at the AlGaN/GaN interface without intentional doping [6,7]. It is generally accepted that the high 2DEG concentration arises from the strong spontaneous and piezoelectric polarization effects of III-nitride (III-N) materials [7,8,9]. However, there is currently no consensus regarding the microscopic origin of the 2DEG in bare AlGaN/GaN heterojunctions.

Many researchers have investigated the origin of the 2DEG in bare AlGaN/GaN heterojunctions and have proposed several mechanisms. The most widely accepted theory is that the 2DEG originates from donor-like states on the AlGaN surface [10,11,12,13,14,15,16,17,18]. This offers a theoretical understanding of the 2DEG origin; however, two questions remain unanswered: what is the type of matter that produces these donor-like states, and why do these states show a specific distribution in the bandgap? The attraction of ions from the ambient atmosphere provides a reasonable explanation [9,19]; however, it does not address the first question. Surface oxidation has also been considered based on calculations [15,20,21,22] and experimental results [22]. Surface oxides are very stable owing to the strong Ga-O bond and are mostly unaffected by the room temperature (~25 °C) environment. However, the 2DEG in AlGaN/GaN heterojunctions without passivation is not very stable. In particular, polar organic liquids on the AlGaN/GaN surface can reduce the 2DEG concentration by 66.7% [19]. These results suggest that the 2DEG has other origins.

Researchers have also shown that the 2DEG in AlGaN/GaN heterojunctions is highly sensitive to polar organic liquids [19]. This high sensitivity is attributed to the direct interactions between the polar liquids and AlGaN surfaces, including surface charge compensation [19,23], surface potential change [24,25,26], and large dipole moment-induced negative charges [27]. However, in this study, we showed that the 2DEG cannot be recovered once the polar liquid is removed, which contradicts the direct polar liquid–AlGaN interaction mechanism.

In this study, we investigate 2DEG variations in a bare AlGaN/GaN heterojunction under different environments and excitations, including polar liquid immersion, vacuum environment, and ultraviolet (UV) excitation, in different atmospheres. First-principles calculations are performed to estimate the states of O_2_ molecules on the AlN and GaN surfaces. Based on the experimental and theoretical results, we present a new model, i.e., “dynamic surface-modulation doping of gas molecules”, to explain the microscopic origin of the 2DEG in bare AlGaN/GaN heterojunctions.

## 2. Results

### 2.1. Effects of Water and Polar Organic Liquids on AlGaN/GaN Heterojunction

**Figure 1:**
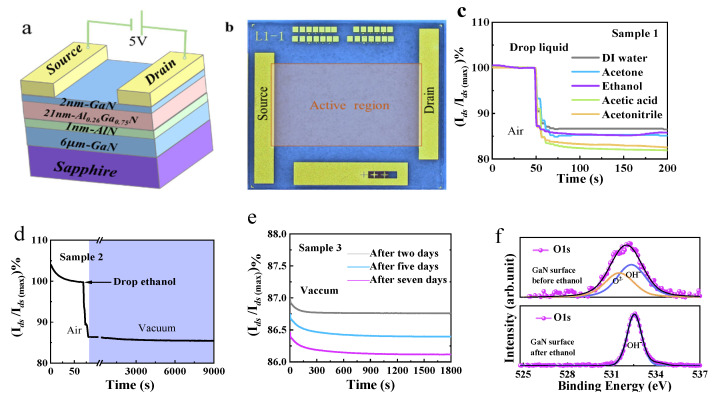
Ungated AlGaN/GaN HEMT in different environments. (**a**) Diagram of the ungated AlGaN/GaN HEMT structure. (**b**) Top view of the ungated AlGaN/GaN HEMT. (**c**) *I_ds_*–*t* curves for different organic liquids dropped onto the surface of the AlGaN/GaN heterojunction. (**d**) *I_ds_*–*t* curve of the device in a dark vacuum chamber (10^−3^ Pa) after ethanol was dropped onto the surface. (**e**) *I_ds_* variations in the device in a dark vacuum chamber (10^−9^ Pa) over one week. (**f**) O1s signal from the device surface before and after the dropwise addition of ethanol obtained via X-ray photoelectron spectroscopy (XPS).

Figure 1a,b show the ungated Al_0.26_Ga_0.74_N/GaN heterojunction used in our experiments. A 2DEG channel was located at the AlN/GaN interface. We applied a bias of *V_ds_* = 5 V between the source and drain electrodes, and the drain–source current (*I_ds_*) in the linear region was used as a direct indicator of the 2DEG concentration (*n_s_*).

Deionized (DI) water, acetone, acetic acid, acetonitrile, and ethanol were dropped separately onto the surface of the ungated AlGaN/GaN HEMT (Sample 1). (For test details, refer to Section S1 of the Supporting Information). *I_ds_* as a function of time is shown in Figure 1c.

In agreement with the results of previous reports [19,23,25], the addition of water and polar organic liquids to the AlGaN/GaN surface reduced the 2DEG concentration and caused *I_ds_* to decrease rapidly by up to ~21.5% (as shown in Figure 1c,d). Therefore, *n_s_* was found to decrease by approximately 20%.

We also dropped ethanol onto Sample 2, placed it in a dark vacuum chamber at 10^−3^ Pa, and recorded *I_ds_* as a function of time; the plot is shown in Figure 1d (see Section S2 of the Supporting Information for further details). When ethanol was added to the sample, *I_ds_* decreased. In a vacuum, ethanol evaporates, and *I_ds_* recovers to the initial value if polar liquids reduce *n_s_* directly. However, *I_ds_* remained low for a long time (~2.5 h) with no sign of recovery. This may be because the polar liquid molecules were persistently adsorbed on the AlGaN/GaN heterojunction owing to the strong polarization of the heterojunction or other unknown mechanisms.

Sample 3 was cleaned sequentially using acetone, isopropanol, and DI water in an ultrasonic bath. Then, it was dried over N_2_ and placed in a dark vacuum chamber at ~10^−9^ Pa (see Section S2 of the Supporting Information for further details). The *I_ds_*–*t* curves were recorded after two, five, and seven days, as shown in Figure 1e. This showed that *I_ds_* decreased continuously over time. If the polar liquid molecules were persistently adsorbed on the AlGaN/GaN heterojunction, then *I_ds_* should remain at a constant, relatively low value over time but should not continue to decrease. Therefore, we concluded that the decrease in *I_ds_* (that is, the decrease in *n_s_*) was caused by the desorption of some components in the atmosphere to which Sample 3 was exposed.

The surface of the AlGaN/GaN heterojunction before and after the addition of ethanol was analyzed through X-ray photoelectron spectroscopy (XPS); the XPS spectra are shown in Figure 1f. When ethanol was added, the intensity of the peak ascribed to O decreased. In particular, the intensity of the peak at approximately 532 eV corresponding to O_2_ was substantially reduced. This evidence also negates the possibility of persistent adsorption of polar organic liquids on the AlGaN/GaN heterojunction, which would increase the intensity of the O peak [28]. Therefore, we conclude that polar liquids do not directly affect *n_s_*. Neglecting other factors that are believed to be the true origins of 2DEG, i.e., the surface cleaning effect, it can be observed that the *I_ds_* of the AlGaN/GaN heterojunction exhibits indirect “sensitivity” to polar liquids. These results provide guidance to deduce the factors that affected the 2DEG concentration. In particular, such factors must be present in the air, must be volatile, and should probably contain O.

### 2.2. Effects of Atmosphere on AlGaN/GaN Heterojunction under UV Illumination

Based on the above results, we tested Sample 4 in a dark environment under air (see Section S2 of the Supporting Information for further details). The current did not recover for a long time (~7 h); this implies that surface adsorption is highly unlikely to occur in the absence of outer excitation. Next, we designed an experiment with UV illumination to test current recovery in different atmospheres.

**Figure 2:**
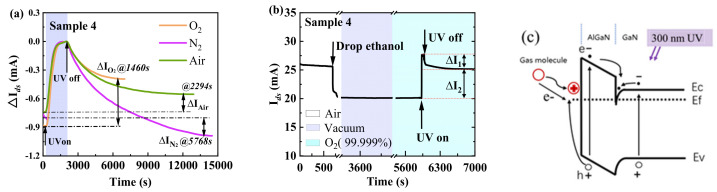
Effects of an ungated AlGaN/GaN HEMT excited by UV light. (**a**) Δ*I_ds_*–*t* curves of the ungated AlGaN/GaN HEMT under low-power UV illumination and in different gas atmospheres. (**b**) *I_ds_*–*t* curve of the ungated AlGaN/GaN HEMT under higher-power UV illumination and in an O_2_ atmosphere. (**c**) Schematic of the energy band of the ungated AlGaN/GaN HEMT under UV illumination and in an O_2_ atmosphere.

Ethanol was dropped onto Sample 4, which was then placed in a dark vacuum chamber. Once the vacuum stabilized at 10^−4^ Pa and the ethanol evaporated, the chamber was ventilated using air, N_2_ (99.999%), or O_2_ (99.999%) (see Section S3 of the Supporting Information for further details). A UV light-emitting diode (LED) with a peak wavelength of 300 nm and power of 0.24 μW was used to illuminate the sample for 1980 s. Once *I_ds_* was approximately saturated, the UV LED was turned off. The Δ*I_ds_*–*t* curves are shown in Figure 2a with initial *I_ds_* (*t* = 0) as a reference. When the UV LED was turned off, *I_ds_* slowly decreased from the peak value to a stable value (decrease in Δ*I_air_*, Δ*I_O2_*, and Δ*I_N2_* in air, O_2_, and N_2_, respectively). It was found that Δ*I_O2_* > Δ*I_air_* > 0 > Δ*I_N2_*. That is, when excited by 300 nm light, O_2_ and air induced additional 2DEG, whereas N_2_ did not. Furthermore, *τ_O2_* ≈ 1460 s < *τ_air_* ≈ 2294 s < *τ_N2_* ≈ 5768 s, where *τ* is the persistent photoconductivity (PPC) decay-time constant [29].

We repeated this experiment in an O_2_ atmosphere with a high UV power of 80 μW for 80 s. As shown in Figure 2b, the stable *I_ds_*@7000 s (I*_ds_* of the sample at 7000 s) of 25.17 mA was very close to the initial *I_ds_*@688 s (I*_ds_* of the sample at 688 s, before ethanol cleaning) value of 25.60 mA, which shows that the 2DEG was approximately recovered. This phenomenon is very similar to the photo adsorption of O on the surface of GaN [30].

To further explain this phenomenon, we plotted a bandgap diagram (Figure 2c), and for easy understanding, we simplified the energy band diagram by assuming that oxygen molecules are directly adsorbed on the surface of AlGaN. When the AlGaN/GaN heterojunction was illuminated by 300 nm light, electron–hole pairs in both the AlGaN barrier layer and GaN buffer were excited. The electrons in the AlGaN barrier and GaN buffer, driven by the inner built-in electric field, drifted into the 2DEG channel, which increased the 2DEG concentration; thus, the current increased accordingly. The holes in the GaN buffer remained there and did not affect the AlGaN surface owing to the valence band barrier at the AlGaN/GaN interface. The holes in the AlGaN barrier layer drifted to the surface and accumulated there. When O_2_ molecules reached the AlGaN surface [30], the holes transferred to the O_2_ molecules, which thereby changed to positive molecule ions and were absorbed on the AlGaN surface. When the illumination was turned off, the photo-induced current was reduced to zero. However, the ions remained on the AlGaN surface, and according to the charge neutrality principle, the same number of electrons were in the 2DEG channel, which resulted in the additional current.

The PPC decay-time constant depends on the hole-consumption rate. In a N_2_ atmosphere, the holes are consumed as they recombine with the 2DEG. In an O_2_ atmosphere, in addition to recombination with the 2DEG, surface holes are consumed as they are transferred to O_2_ molecules. The hole-consumption rate in an O_2_ atmosphere was higher than that in a N_2_ atmosphere. Therefore, the PPC decay-time constant in an O_2_ atmosphere (*τ_O2_*) was lower than that in a N_2_ atmosphere (*τ_N2_*). Notably, the stable current in a N_2_ atmosphere was lower than the initial value (Δ*I_N2_* < 0), possibly owing to the N_2_ atmosphere carrying some O_2_ ions from the AlGaN surface.

From this observation, the critical question arises as to why air and O_2_ induce the observed effects, whereas N_2_ does not. As indicated by the semiconductor theory, the electrons can occupy or transfer in/out of the material only in an energy state. Therefore, we next considered which gas molecules could have an energy state and the depth of the energy state on the III-N material surface.

### 2.3. First-Principles Calculation

Previously, some researchers have conducted first-principles calculations and suggested that the Ga-O bond on the AlGaN surface is the origin of the 2DEG in the AlGaN/GaN heterojunction [20]. Our vacuum experiments (see above) also suggested that the adsorption matter is volatile. The Ga-O bond has a high formation energy of −10.4 eV and is very difficult to break at room temperature (~25 °C), thereby imparting non-volatile properties [31]. Furthermore, there is no evidence that polar liquids can remove O from the AlGaN surface by breaking the Ga-O bond. Therefore, we focused on investigating the interactions between the gas molecules and III-N material using first-principles calculations, i.e., the possibility of a bond with an adsorption energy stronger than physical adsorption while weaker than chemical adsorption.

**Figure 3:**
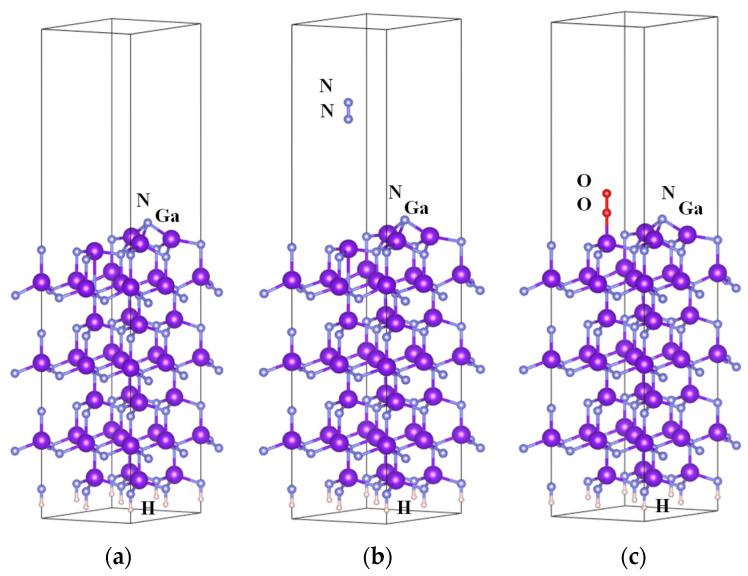
Side views of the optimized GaN(0001) structures. (**a**) N_H3_ adatom reconstruction, (**b**) N_2_ on the surface, and (**c**) O_2_ adsorbed on the surface.

We used the density functional theory (DFT) to calculate the adsorption energies of N_2_ and O_2_. N_H3_ adatom reconstruction, shown in Figure 3a, was performed to satisfy the electron counting rule. The adsorption energy is defined as Δ*E_ad_* = *E_ad_* − (*E_sur_* + *E_gas_*). For N_2_, Δ*E_ad_* = 0.112 eV. Δ*E_ad_* is unstable for N_2_ adsorbed on the GaN surface; therefore, N_2_ was far from the surface, as shown in Figure 3b. For O_2_, Δ*E_ad_* = −0.461 eV. Δ*E_ad_* is stable for O_2_ adsorbed on the GaN surface, as shown in Figure 3c; therefore, O_2_ could still be adsorbed after a certain rotation of ~30°.

Since van der Waals forces act between the molecule and the surface, we switch on the use of the original vdW-DF functional [32]. These functionals depend on the electron density at two points in space and model long-range van der Waals (dispersion) correlation effects. For N_2_, Δ*E_ad_* = −0.266 eV. N_2_ molecules are adsorbed onto the surface. For O_2_, Δ*E_ad_* = −0.636 eV. Δ*E_ad_* is more stable for O_2_ adsorbed on the GaN surface.

**Figure 4:**
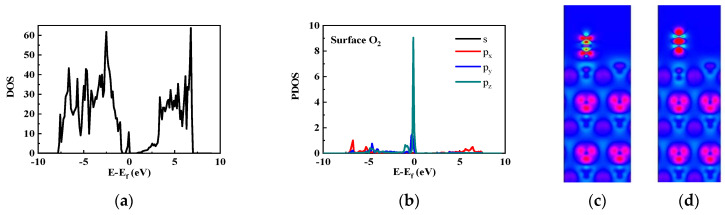
(**a**) DOS, (**b**) PDOS, and the deformation electron density of (**c**) O_2_ and (**d**) N_2_ adsorbed on the GaN(0001) surface.

We studied the density of states (DOSs) and partial density of states (PDOSs) of O_2_ adsorbed on the GaN(0001) surface; the results are shown in Figure 4a and b, respectively. O_2_ formed an energy level at the Fermi energy. From the PDOS of a surface O atom, we found that the electrons at this energy level originated from the O p orbital. Based on Heyd–Scuseria–Ernzerhof (HSE) calculations, we determined the valence band maximum (VBM) energy, conduction band minimum (CBM) energy, defect energy of N_H3_, and defect energy of surface O atoms to be −1.745, 1.208, −0.283, and 0.108 eV, respectively.

Next, we investigated the effects of O_2_ bonding on the GaN(0001) surface by computing the deformation electron density. For O_2_ on GaN(0001), the deformation electron density can be expressed as Δ*ρ* = *ρ_O2@GaN(0001)_* − Σ *ρ_i_*, where *ρ_O2@GaN(0001)_* is the deformation electron density of the total O_2_ + GaN(0001) system, and *ρ_i_* is the electron density of the *i*_th_ atom. We considered a slide-through O_2_ molecule and Ga atom, as shown in Figure 4c (where increases and decreases in electron density are indicated by red and blue regions, respectively). As a comparison, the deformation electron density of the N_2_ + GaN(0001) system is shown in Figure 4d. The figures indicate that a weak bond existed between the O and Ga atoms, the deformation electron density of the Ga atom increased slightly, and little electron transfer occurred from O_2_ to Ga. But, there is no similar bond or charge transfer between Ga and N_2_.

It is well known that in AlGaN/GaN heterojunctions, the AlGaN barrier layer must exceed the critical thickness to generate the 2DEG. Based on the first-principles calculations, the energy level of O_2_ was estimated to be 1.391 eV below the CBM of the GaN surface, which resulted in a critical barrier thickness of 3.02 nm for the Al_0.26_Ga_0.74_N/GaN heterojunctions. This is consistent with the results reported in previous reports [15].

The surface energy level of O_2_ on AlN was determined to be 3.56 eV below the CBM by first-principles calculation. Therefore, it is reasonable to generalize the energy levels of O_2_ on Al_x_Ga_1−x_N (0 ≤ *x* ≤1), the Al, Ga, N ternary compound with arbitrary Al composition of x (see Section S4 of the Supporting Information for further details). This may help people estimate the bandgap diagram of the bare Al_x_Ga_1−x_N/GaN heterojunctions.

### 2.4. Physical Model: Gas–Molecule Dynamic Modulation Doping on AlGaN/GaN Heterojunction

Following the first-principles calculations, we concluded that some types of gas molecules have energy states on the III-N surface. When these molecules collide with the III-N surface, electrons can be transferred from the molecules to the 2DEG channel, which causes the positively charged molecules to be adsorbed on the surface. The amount of adsorbed molecules depends on the inner polarization field (driving force) and environment (outside condition). The adsorption and desorption of molecules occur constantly, and under stable conditions, they reach an equilibrium state with a relatively stable 2DEG concentration. The equilibrium state is sensitive to environmental conditions. We verified this observation using O_2_ as an example.

**Figure 5:**
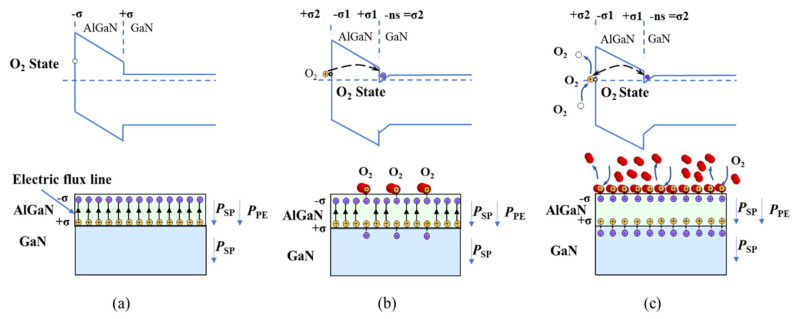
Schematic cross-sectional and bandgap diagrams of the physical model of dynamic modulation doping generated by O_2_ adsorption on the AlGaN/GaN surface. (**a**) Clean surface. (**b**) O_2_ molecules adsorbed on the AlGaN/GaN surface. (**c**) Adsorption of O_2_ molecules at equilibrium on the AlGaN/GaN surface.

As shown in Figure 5a, for a fresh AlGaN/GaN heterojunction with an AlGaN barrier layer that is thicker than the critical thickness, sheets of positive +σ and negative −σ fixed charges are induced by spontaneous and piezoelectric polarizations at the interface and surface, respectively. The positive and negative fixed charges are coupled to each other in pairs, and there is no 2DEG in the AlGaN/GaN interface channel. The O_2_ molecules on the AlGaN surface have energy states above the Fermi energy level; however, because there are no O_2_ molecules in the environment, those O_2_ energy states do not function.

As shown in Figure 5b, when there are O_2_ molecules in the environment, they contact the AlGaN surface, and the O_2_ energy states assist the transfer of electrons from the O_2_ molecules to the 2DEG channel. Thus, the initial fixed charge coupling (+σ and −σ) is broken. Subsequently, new charge couplings are established; the positively charged O_2_ molecules couple with the same number of negative fixed charges on the surface, and the electrons in the channel couple with the same number of positive fixed charges at the interface. Under these charge couplings, the positively charged O_2_ molecules adsorb on the AlGaN surface in a higher stable state than those undergoing pure physical adsorption. As O_2_ models collide and transfer electrons, more O_2_ ions are adsorbed on the AlGaN surface, and more electrons enter the 2DEG channel. This continues until the O_2_ energy states and surface potential drop to the Fermi energy level.

As shown in Figure 5c, the adsorption of O_2_ molecule ions is a dynamic process, which indicates that adsorption and desorption occur simultaneously and continuously. If the adsorption rate is higher than the desorption rate, the 2DEG concentration increases. If the adsorption rate is lower than the desorption rate, the 2DEG concentration decreases. When the adsorption rate is equal to the desorption rate, equilibrium is established, and the 2DEG concentration is stable.

During the O_2_ adsorption process, there is a barrier height that blocks the transfer of electrons from the O_2_ molecule to the channel by reducing the transfer probability, which would be accompanied by a very low adsorption rate. This was verified by the results of the experiment conducted under dark conditions and in an air atmosphere (see Section S2 of the Supporting Information for further details). Additional excitation, as under UV illumination, can increase the adsorption rate, as shown in Figure 2.

In general, difficult adsorption corresponds to easy desorption. However, it appears that this rule does not apply to the AlGaN/GaN heterojunction, as demonstrated by the findings of the vacuum experiment shown in Figure 1e. This results from the re-established dipole coupling between the positive molecule ion and negative fixed charge at the surface. The dipole moment is very small owing to the short distance; therefore, the molecule ion adsorption exhibits higher stability than general physical adsorption.

## 3. Conclusions

In this study, we investigated the interactions between various molecules and an AlGaN/GaN heterojunction, unlike previous studies that focus only on the interactions between atoms and AlGaN/GaN heterojunctions, to determine the microscopic origin of the 2DEG in bare AlGaN/GaN heterojunctions. Thus, a new mechanism was proposed—the dynamic surface modulation doping effect of gas molecules on AlGaN/GaN heterojunctions. This mechanism effectively explains the microscopic origin of the 2DEG and shows that the sensitivity of AlGaN/GaN heterojunctions to polar liquids is determined by the surface cleaning effect. This study provides insights into III-N materials and promotes the development of new device fabrication processes and novel devices.

## Data Availability

The authors confirm that the data supporting the findings of this study are available within the article and its Supplementary Materials.

## Supplementary Materials

The following supporting information can be downloaded at: https://www.mdpi.com/article/10.3390/nano14141211/s1, References [33,34,35,36,37,38,39,40,41,42,43,44,45] are cited in the supplementary materials.
