# Supplementary material for: The Dynamic Modulation Doping Effect of Gas Molecules on an AlGaN/GaN Heterojunction Surface
Source: Nanomaterials (Basel). 2024 Jul 16;14(14):1211. doi: 10.3390/nano14141211 (PMC11280321; doi:10.3390/nano14141211)
Supplement: Supplementary file 1 [file nanomaterials-14-01211-s001.zip › nanomaterials-3011991-supplementary.pdf]

Supplementary Material

The dynamic-modulation doping effect of gas molecules on an AlGaN/GaN heterojunction surface

Section 1: Sample fabrication and electrical testing condition

The epi-layers were grown on a sapphire substrate via metalorganic chemical vapor deposition (MOCVD). From bottom to top, they consisted of a 6 μm thick unintentional doped GaN buffer layer, 1 nm thick AlN interlayer, 21 nm thick $Al_{0.26}Ga_{0.74}N$ barrier, and 2 nm thick GaN cap layer. Mesa isolation was performed using Cl_2/BCl_3 inductively coupled plasma reactive ion etching (ICP-RIE). The Ohmic contacts were fabricated via e-beam evaporation of Ti/Al/Ni/Au (20/130/50/100 nm) stacks and then annealed at 870 °C for 30 s in a N_2 atmosphere. After device fabrication and dicing, the 5 ×5 mm^2 chips were mounted on a printed circuit board (PCB).

The *L/W* ratios of Samples 1–4 are shown in Table S1.

TABLE S1. Geometry of the samples.

Sample ($Al_{0.26}Ga_{0.74}N$/GaN)	*L* (μm)	*W* (μm)	*L*/W
Sample 1	1600	2200	0.73
Sample 2	100	400	0.25
Sample 3	3800	2500	1.52
Sample 4	2050	3000	0.68

Note: *W* is the width of the active region, and *L* is the distance between the drain and source.

The *I–V* characteristics of Sample 4 are shown in Fig. S1. To avoid the trapping effect under high electric fields, we conducted the tests in the linear region of the *I–V* curve.

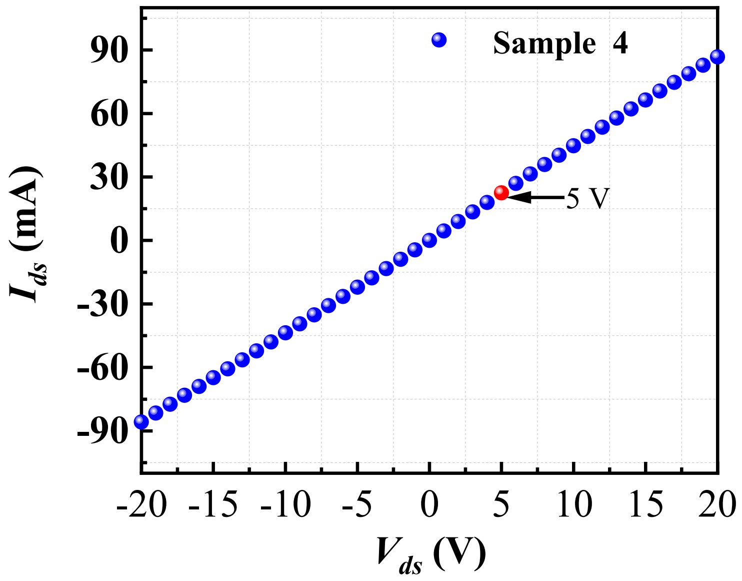

FIG. S1. *I–V* characteristics of Sample 4.

A voltage of 5 V was applied to both pads of the source/drain electrodes every 1 s, and the corresponding current was recorded. This method prevented thermal current effects.

For an ungated AlGaN/GaN high-electron-mobility transistor (HEMT), the drain–source current (I_{ds}) in the linear region of the *I–V* curve can be expressed as follows:

$$I_{ds} = qV_{ds}W/L\mu(T)n_s, \quad (1)$$

where q is the electrostatic charge, V_{ds} is the bias voltage, W is the width of the active region, L is the distance between the drain and source, $\mu(T)$ is the electron mobility that is affected by the temperature, and n_s is the two-dimensional electron gas (2DEG) sheet concentration. Thus, I_{ds} mainly depends on n_s when the temperature variation is very small. Hence, at a fixed V_{ds}, I_{ds} can be taken as a direct indicator of n_s. In the experiments, V_{ds} was 5 V, which is within the linear region of the I–V curve as shown in Fig. S2. As discussed in the main text, some I_{ds}–t curves were normalized to obtain the relative variations. The absolute I_{ds}–t curves are shown below.

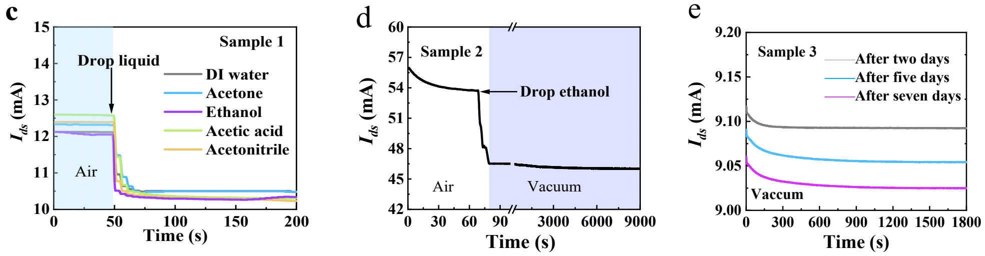

FIG. S2. Effects of an ungated AlGaN/GaN HEMT in different environments. (c) I_{ds}–t curves with different organic liquids dropped onto the AlGaN/GaN heterojunction surface. (d) I_{ds}–t curve of the device in a dark vacuum (10^{-3} Pa) chamber after ethanol was dropped onto the surface. (e) I_{ds} variations of the device in a dark vacuum chamber (10^{-9} Pa) over one week.

Section 2: Testing procedures in different environments

Sample 1 was exposed to air, a bias of 5 V was applied every 1 s to the top of the source/drain pads, and I_{ds} was recorded. When the current stabilized, an organic liquid was dropped onto the surface of the exposed active area and I_{ds} was recorded again. The results are shown in Fig. 1(c) in the main text. After the test, the sample was placed in a light environment and the concentration 2DEG was allowed to recover to the original value before testing the next liquid.

Sample 2 was prepared by dropping ethanol onto the surface; then, it was placed in a vacuum chamber and evacuated to 10^{-3} Pa. I_{ds} was recorded continuously for ~2.5 h; the results are shown in Fig. 1(d) in the main text.

Sample 3 was cleaned sequentially using acetone, isopropanol, and DI water. Then, it was placed in the vacuum chamber of a low-temperature nanoprobe with a scanning tunneling microscope (SenticaOmicron). The vacuum was maintained at 2.5×10^{-9} Pa, and the I_{ds} of the sample was tested in the vacuum chamber at 48, 120, and 168 h. A V_{ds} of 5 V was applied for 1800 s, and the I_{ds}–t curve was recorded. The results are shown in Fig. 1(e) of the main text.

Sample 4 was prepared by dropping ethanol onto the surface; then, it was exposed to an air atmosphere overnight and I_{ds} was recorded; the I_{ds}–t curve is shown in Fig. S3. There were no signs of recovery of I_{ds} after 7 h. After sunrise, the current increased slowly (the sample was not directly exposed to sunlight). At noon, the current increased rapidly. Compared with the experimental conditions for Sample 1, we found that light was a key factor for I_{ds} recovery in the ungated $Al_{0.26}Ga_{0.74}N$/GaN HEMT. Therefore, we conducted ultraviolet (UV) excitation experiments with different gas atmospheres to identify which gases aided current recovery.

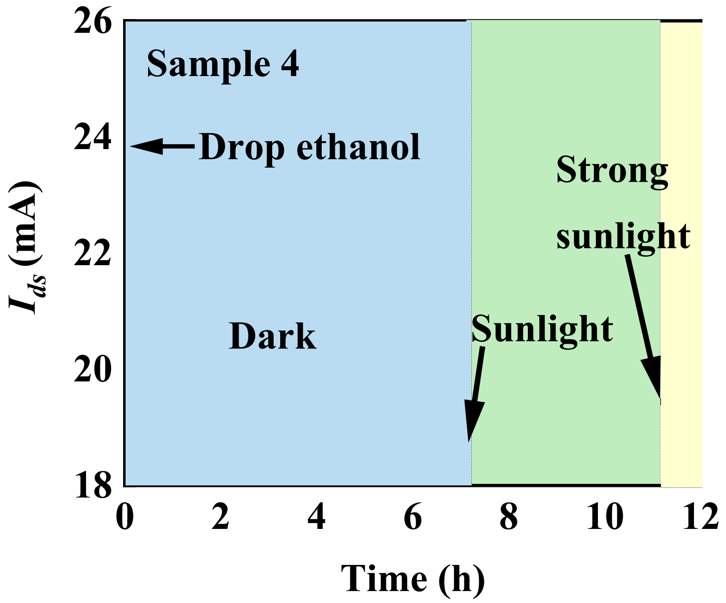

FIG. S3. I_{ds}–t curve for Sample 4 in an air atmosphere overnight.

Section 3: UV excitation experiments in different gas atmospheres

For the UV excitation experiments in different gas atmospheres, a Turbolab vacuum pump (Leybold) was used to maintain a vacuum of 10^{-4} Pa. The O_2 and N_2 gases had purities of 99.999%. A UV-light emitting diode (LED; 300 nm ± 10%; Shenzhen Lamplic Science Co., Ltd.) with an output power of 0.24 μW was used as the light source. A schematic of the experimental setup is shown in Fig. S4. The vacuum chamber was enclosed in several layers of aluminum foil to block external light.

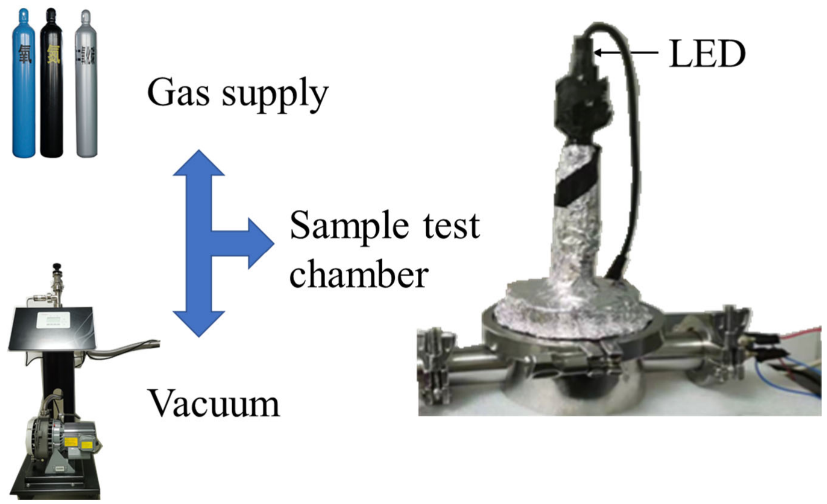

FIG. S4. Setup of the experiment conducted to verify the effect of the gas atmosphere on the concentration of the 2DEG in the AlGaN/GaN heterojunction.

First, the surface of Sample 4 was cleaned using an anhydrous ethanol solution; then, the vacuum chamber was pumped to ~10^{-4} Pa. Once the current stabilized, the vacuum pump valve was closed and a high-purity gas was introduced rapidly into the vacuum chamber while the negative pressure inside the chamber was maintained. The UV LED was then turned on. Once I_{ds} was approximately saturated, the UV LED was turned off. I_{ds} was recorded until it was approximately stable. The heat generated by UV light irradiation on Sample 4 gradually dissipated after the light was turned off, and the temperature of Sample 4 returned to the same value as that

before the UV light irradiation. The change in current was caused by the change in 2DEG concentration. The results are shown in Fig. 2(a) of the main text.

To verify the effect of the O_2 gas on the 2DEG concentration in the AlGaN/GaN heterojunction, we increased the power of the UV LED to 0.08 mW and repeated the experiment in an O_2 atmosphere. The I_{ds} with a bias of 5 V was recorded from the beginning of the experiment until it stabilized. The measured I_{ds}–t curve is shown in Fig. 2(b) of the main text.

The stable I_{ds} of 25.17 mA was approximately the same as the initial value of 25.60 mA, which suggests that there was sufficient 2DEG recovery. Figure 2(b) of the main text shows that the I_{ds}–t curve after the UV LED was turned off was consistent with the decay curves. Thus, ΔI_1 was attributed to the photoconductivity effect, whereas ΔI_2 was attributed to the adsorption of gas molecules.

The decay of the persistent photoconductivity (PPC) followed a stretched exponential function[1-3]. That is,

$$I_{PPC}(t) = I_{PPC}(0)\ \exp[-(t/\tau)^{\beta}],\ 0 < \beta < 1, \qquad (2)$$

where $I_{PPC}(0)$ is the PPC buildup at the moment when the UV LED was switched off, τ is the PPC decay time constant, and β is the decay exponent.

In O_2, air, and N_2 atmospheres, least-squares fits of the experimental data yielded time constants of approximately 1460, 2294, and 5768 s, respectively, and decay exponents of approximately 0.9606, 0.8626, and 0.7358, respectively.

Section 4: First-principles calculations

Density functional theory (DFT) calculations were conducted using fully periodic plane-wave calculations in the Vienna Ab initio Simulation Package (VASP)[4]. Electron exchange and correlation were described using the PW91 generalized gradient approximation (GGA)[5,6], and projector augmented wave function (PAW) methods[7,8] were also used. The subsequent procedures were performed within the Heyd–Scuseria–Ernzerhof (HSE) screened hybrid functional, which included identification of the band structures. To obtain reasonable structures, the energy cut-off was set to 600 eV and the irreducible Brillouin zone grid used a 5 × 5 × 1 mesh. The energy convergence criterion was 1×10^{-5} eV and the atomic-force convergence criterion was -1×10^{-2} eV. The calculated equilibrium lattice parameters of the GaN bulk were a = 3.177 Å, c = 5.167 Å, and u = 0.377. These values agree well with the experimental results where a = 3.20 Å, c = 5.22 Å, and u = 0.3777[9]. Using 28% of the exact exchange (mixing parameter α = 0.28), we determined that the bandgaps of the GaN bulk were 3.48 eV.

Supercell slab models along the (0001) orientation were constructed for the surface calculations based on 4-atom (2 × 2) surface unit cells, and a vacuum of approximately 15 Å between any two slabs was used. The supercell slab included seven monolayers (MLs) with the top two MLs relaxed. The N dangling bonds on the $(000\bar{1})$ surface at the back of the slabs were passivated by fractionally charged H atoms with a charge of 0.75. N_{H3} adatom reconstruction was performed to satisfy the electron counting rule.

The different initial rotation angles were determined as follows:

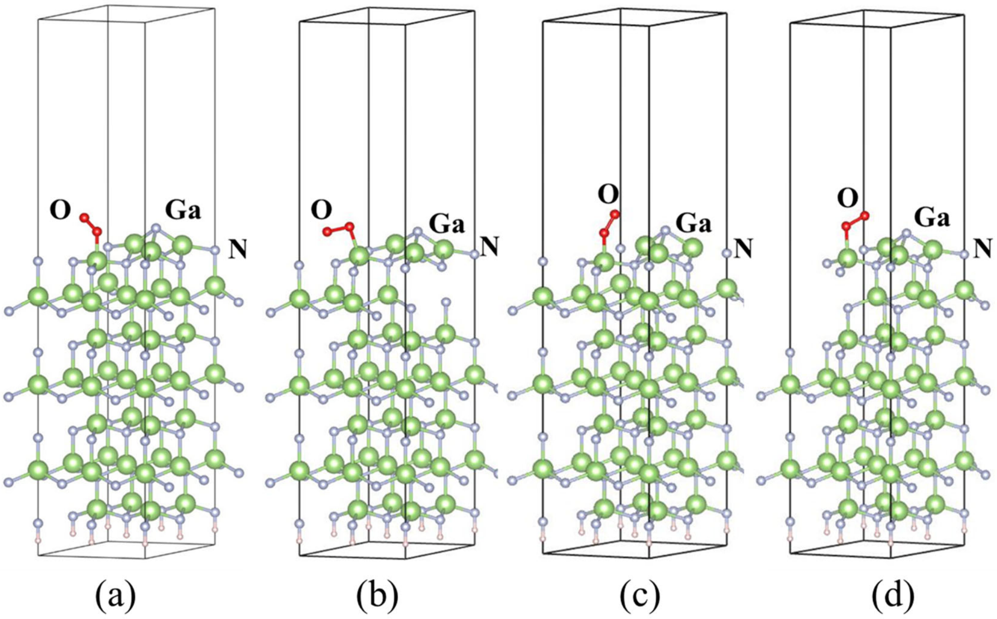

FIG. S5. Side view of the structures with O_2 adsorbed on GaN(0001) with different initial rotation angles. $\theta = 30°$ and $\varphi = 0°$ (a) before and (b) after relaxation. $\theta = 30°$ and $\varphi = 180°$ (c) before and (d) after relaxation.

For $\theta = 30°$ and $\varphi = 0°$, the adsorption energies were determined to be $\Delta E_{ad} = -1.472$ eV. For $\theta = 30°$ and $\varphi = 180°$, the adsorption energies were determined to be $\Delta E_{ad} = -1.000$ eV. O_2 could still be adsorbed after a certain rotation.

We also calculated the related properties of AlN. The calculated equilibrium lattice parameters of the AlN bulk were $a = 3.097$ Å, $c = 4.955$ Å, and $u = 0.382$. These results agree well with the experimental results where $a = 3.11$ Å, $c = 4.98$ Å, and $u = 0.382$[10]. Using 32% of the exact exchange (mixing parameter $\alpha = 0.32$), we determined that the bandgaps of the AlN bulk were 6.07 eV.

Using DFT calculations, we determined the adsorption energies of O_2 on the AlN(0001) surface. The N_{H3} adatom reconstruction was performed to satisfy the electron counting rule, as shown in Fig. S6(a). For O_2, $\Delta E_{ad} = -0.877$ eV; ΔE_{ad} is stable for O_2 adsorbed on the AlN surface, as shown in Fig. S6(b).

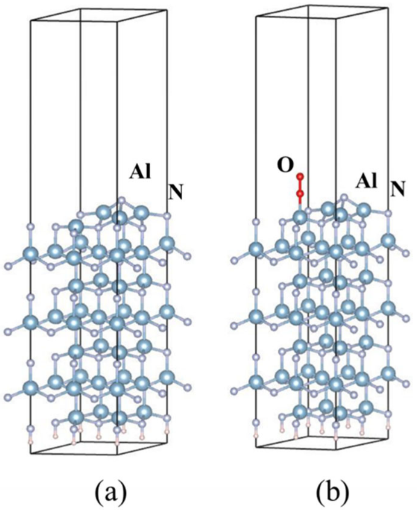

FIG. S6. Side view of the optimized structures of AlN(0001). (a) N_{H3} adatom reconstruction and (b) O_2 adsorbed on the surface.

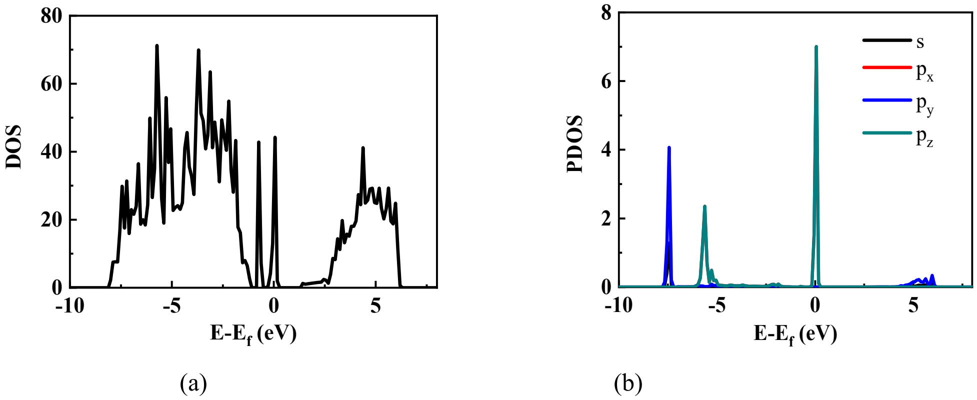

FIG. S7. (a) Density of states (DOS) and (b) partial density of states (PDOS) of O_2 absorbed on the AlN(0001) surface.

We studied the DOS and PDOS of O_2 absorbed on the AlN(0001) surface; the results are shown in Fig. S7(a) and (b). The O_2 formed an energy level at the Fermi energy. From the PDOS, we determined that the electrons in the energy level at the Fermi energy originated from the O p orbital. Based on the HSE calculations, we determined the valence band maximum (VBM) energy, conduction band minimum, defect energy of N_{H3}, and defect energy of surface O atoms to be −2.415, 2.723, −1.978, and −0.774 eV, respectively.

To estimate the energy levels of O_2 on the surface of AlxGa1-xN ($0 \leq x \leq 1$), we performed linear interpolation[11]; the results are shown in Fig. S8. The VBMs of GaN and AlN were aligned according to the results reported by Canedy et al.[12], and bandgap bowing was neglected.

The bandgaps calculated for GaN and AlN (2.96 and 6.07 eV, respectively) deviated slightly from the real bandgaps (3.4 and 6.2 eV, respectively); therefore, the energy levels for O_2 ($E_{O2\text{-}GaN}$ and $E_{O2\text{-}AlN}$) were determined using the following equations:

$$E_{O2\text{-}GaN} = E^*_{O2\text{-}GaN} \times E_{GaN}/E^*_{GaN} \quad (3)$$

and

$$E_{O2\text{-}AlN} = E^*_{O2\text{-}AlN} \times E_{AlN}/E^*_{AlN}, \quad (4)$$

where $E^*_{O2\text{-}GaN}$ and $E^*_{O2\text{-}AlN}$ are the calculated energy levels of O_2 on the surfaces of GaN and AlN, respectively, and E^*_{GaN} and E^*_{AlN} are the calculated bandgaps of GaN and AlN.

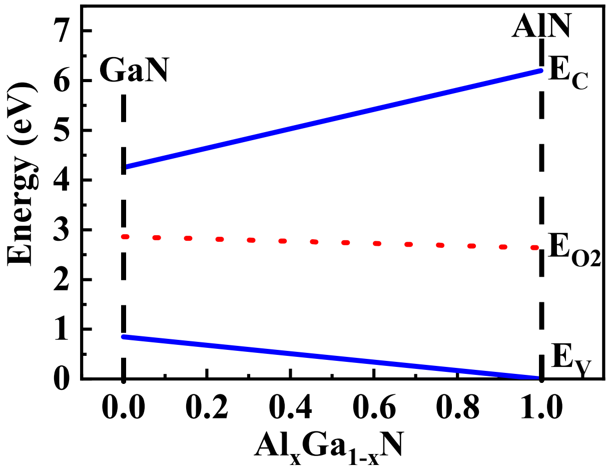

FIG. S8. Interpolation of the position of the occupied O_2 surface state for $Al_xGa_{1-x}N$ alloys.

Section 5: Estimation of critical AlGaN barrier thickness

As the AlGaN barrier thickness increases, the polarization field causes the surface potential to increase until it reaches a critical point where the occupied surface donor levels align with the CBM of GaN[13]. According to the first-principles calculations presented in the main paper, the energy level of O is 1.391 eV below the CBM of the GaN surface.

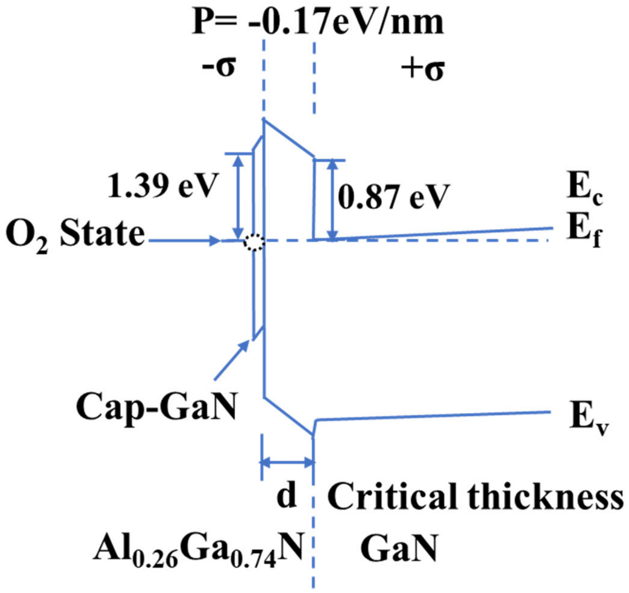

FIG. S9. Schematic diagram showing the O_2 energy levels on the surface of the $Al_{0.26}Ga_{0.74}N$/GaN heterojunction.

$Al_{0.26}Ga_{0.74}N$/GaN has an edge discontinuity band order of $\Delta E_c = 0.7\cdot\Delta E_g$, where ΔE_g is calculated using the following equations[14]:

$$\Delta E_g = \Delta E_{Al_xGa_{(1-x)}N} - \Delta E_{GaN} \quad (5)$$

where

$$\Delta E_{Al_xGa_{(x-1)}N} = x\cdot\Delta E_{AlN} + (1-x)\cdot\Delta E_{GaN}. \quad (6)$$

The polarization electric field inside the $Al_{0.26}Ga_{0.74}N$ potential barrier layer was calculated as follows[15]:

$$P_{PE}(Al_xGa_{1-x}N/GaN) = -0.0525X + 0.0282(1-X),\ (C/m^2) \quad (7)$$

and

$$P_{SP}(\mathrm{Al_xGa_{1-x}N}) = -0.09X - 0.034(1 - X) + 0.021X(1 - X), \ (\mathrm{C/m^2}). \qquad (8)$$

The introduction of the GaN cap layer increases the effective potential barrier height of the AlGaN layer; however, its surface potential barrier still decreases when a heterojunction is formed with $Al_{0.26}Ga_{0.74}N$. In this calculation, we ignored the effect of the GaN cap layer on the surface potential. Considering the presence of the AlN insertion layer at the $Al_{0.26}Ga_{0.74}N$/GaN interface and the polarization effect, the insertion layer can improve the effective conduction band order of the $Al_{0.26}Ga_{0.74}N$ barrier and GaN channel layers. The insertion of a 1 nm thick layer of AlN can improve the conduction band order by 0.38 eV[16].

Through calculation, it can be shown that when the O_2 energy level is 1.39 eV below the surface GaN conduction band, the corresponding critical thickness is 3.02 nm. This result is consistent with the reported experimental results[17]. Thus, it is reasonable to deduce that the O_2 molecule adsorbed on the surface of $Al_{0.26}Ga_{0.74}N$/GaN is an important microscopic origin of the 2DEG.

References

[1] J. Z. Li, J. Y. Lin, H. X. Jiang, M. A. Khan, Q. Chen, Persistent photoconductivity in a two-dimensional electron gas system formed by an AlGaN/GaN heterostructure, J. Appl. Phys. 82 (1997) 1227–1230. https://doi.org/10.1063/1.365893.

[2] T. Y. Lin, H. M. Chen, M. S. Tsai, Y. F. Chen, F. F. Fang, C. F. Lin, G. C. Chi, Two-dimensional electron gas and persistent photoconductivity in AlxGa1-xN/GaN heterostructures, Phys. Rev. B 58 (1998) 13793–13798. https://doi.org/10.1103/PhysRevB.58.13793.

[3] B. K. Li, W. K. Ge, J. N. Wang, K. J. Chen, Persistent photoconductivity and carrier transport in AlGaN/GaN heterostructures treated by fluorine plasma, Appl. Phys. Lett. 92 (2008) 082105. https://doi.org/10.1063/1.2888743.

[4] G. Kresse, J. Furthmuller, Efficient iterative schemes for ab initio total-energy calculations using a plane-wave basis set, Phys. Rev. B 54 (1996) 11169–11186. https://doi.org/10.1103/PhysRevB.54.11169.

[5] J. A. White, D. M. Bird, Implementation of gradient-corrected exchange-correlation potentials in Car-Parrinello total-energy calculations, Phys. Rev. B 50 (1994) 4954–4957. https://doi.org/10.1103/physrevb.50.4954.

[6] J. P. Perdew, J. A. Chevary, S. H. Vosko, K. A. Jackson, M. R. Pederson, D. J. Singh, C. Fiolhais, Erratum: Atoms, molecules, solids, and surfaces: Applications of the generalized gradient approximation for exchange and correlation, Phys. Rev. B 48 (1993) 4978. https://doi.org/10.1103/physrevb.48.4978.2.

[7] G. Kresse, D. Joubert, From ultrasoft pseudopotentials to the projector augmented-wave method, Phys. Rev. B 59 (1999) 1758–1775. https://doi.org/10.1103/PhysRevB.59.1758.

[8] J. Heyd, G. E. Scuseria, M. Ernzerhof, Hybrid functionals based on a screened Coulomb potential, J. Chem. Phys. 118 (2003) 8207–8215. https://doi.org/10.1063/1.1564060.

[9] H. Schulz, K. H. Thiemann, Crystal-structure refinement of AlN and GaN, Solid State Commun. 23 (1977) 815–819. https://doi.org/Doi 10.1016/0038-1098(77)90959-0.

[10] A. F. Wright, J. S. Nelson, Consistent structural properties for AlN, GaN, and InN, Phys. Rev. B 51 (1995) 7866–7869. https://doi.org/10.1103/physrevb.51.7866.

[11] M. S. Miao, J. R. Weber, C. G. Van de Walle, Oxidation and the origin of the two-dimensional electron gas in AlGaN/GaN heterostructures, J. Appl. Phys. 107 (2010) 123713. https://doi.org/10.1063/1.3431391.

[12] C. L. Canedy, W. W. Bewley, C. S. Kim, M. Kim, I. Vurgaftman, J. R. Meyer, Dependence of type II "W" mid-infrared photoluminescence and lasing properties on growth conditions, J. Appl. Phys. 94 (2003) 1347–1355. https://doi.org/10.1063/1.1586974.

[13] L. Gordon, M. S. Miao, S. Chowdhury, M. Higashiwaki, U. K. Mishra, C. G. Van de Walle, Distributed surface donor states and the two-dimensional electron gas at AlGaN/GaN heterojunctions, J. Phys. D: Appl. Phys. 43 (2010) 505501. https://doi.org/10.1088/0022-3727/43/50/505501.

[14] D. Delagebeaudeuf, N. T. Linh, Metal-(n) AlGaAs-GaAs two-dimensional electron gas FET, IEEE Trans. Electron Devices 29 (1982) 955–960. https://doi.org/10.1109/T-Ed.1982.20813.

[15] Ambacher, O. et al., Two-dimensional electron gases induced by spontaneous and piezoelectric polarization charges in N- and Ga-face AlGaN/GaN heterostructures, J. Appl. Phys. 85 (1999) 3222–3233. https://doi.org/10.1063/1.369664.

[16] R. Coffie, Y. C. Chen, I. Smorchkova, M. Wojtowicz, Y. C. Chou, B. Heying, A. Oki, Impact of ALN interlayer on reliability of AlGaN/GaN HEMTs, in 2006 IEEE International Reliability Physics Symposium Proceedings (2006).

[17] M. Higashiwaki, S. Chowdhury, M. S. Miao, B. L. Swenson, C. G. Van de Walle, U. K. Mishra, Distribution of donor states on etched surface of AlGaN/GaN heterostructures, J. Appl. Phys. 108 (2010) 063719. https://doi.org/10.1063/1.3481412.
